# Tuberculous scleritis in a young Asian Indian girl—a case presentation and literature review

**DOI:** 10.1186/s12348-019-0192-9

**Published:** 2019-12-23

**Authors:** Lagan Paul, Manisha Agarwal, Shalini Singh, Prashant Katre, Aman Sumeet Arora

**Affiliations:** 1 Department of Vitreoretina Services, Dr. Shroff’s Charity Eye Hospital, 5027, Kedarnath Road, Daryaganj, New Delhi, 110002 India; 2 Satguru Eye and Dental Hospital, Kapurthala, Punjab India

## Abstract

Scleritis is the severe painful inflammation of the sclera, which can be infectious or non-infectious. Tuberculosis (TB) is one of the common causes of infectious scleritis. TB, though endemic in countries like India, is rare in pediatric age group. We report a case of a 9-year-old female child who presented with bilateral non-necrotizing anterior scleritis with unilateral posterior scleritis secondary to TB. To our knowledge, this is a rare presentation in pediatric age group, and she is the youngest of few cases of tuberculous posterior scleritis reported in literature.

## Case report

**Fig. 1:**
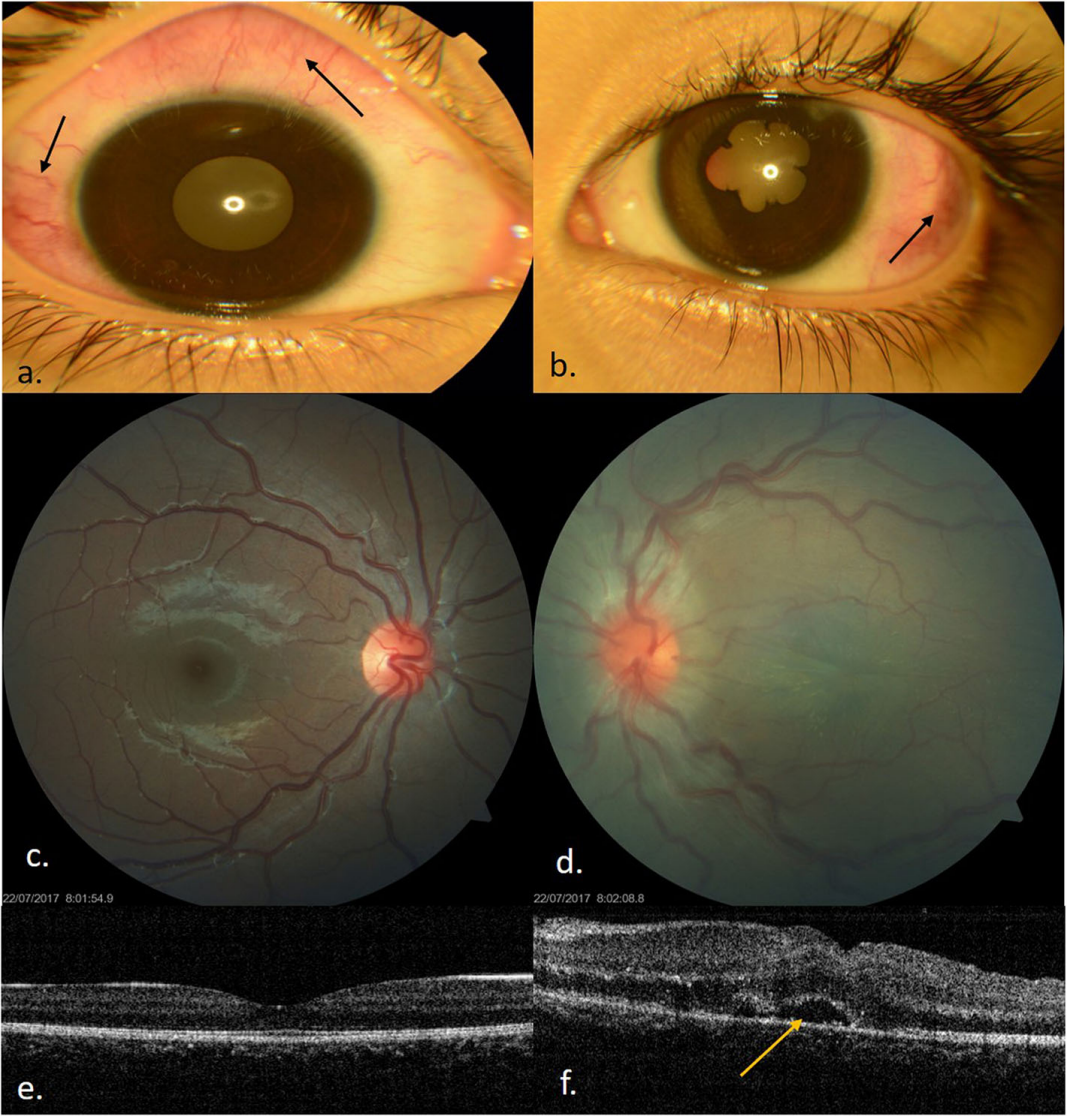
At presentation. **a**, **b** Anterior segment photo of both eyes showing acute nodular anterior scleritis (shown as arrow marks) with posterior synechiae in left eye. **c** Fundus photograph of right eye showing normal fundus. **d** Fundus photograph of left eye showing disc edema with retinal striae and tortous vessels. **e**, **f** OCT of both eyes. Normal in right eye whereas neurosensory detachment with thickening of inner retinal layers in left eye

**Fig. 2:**
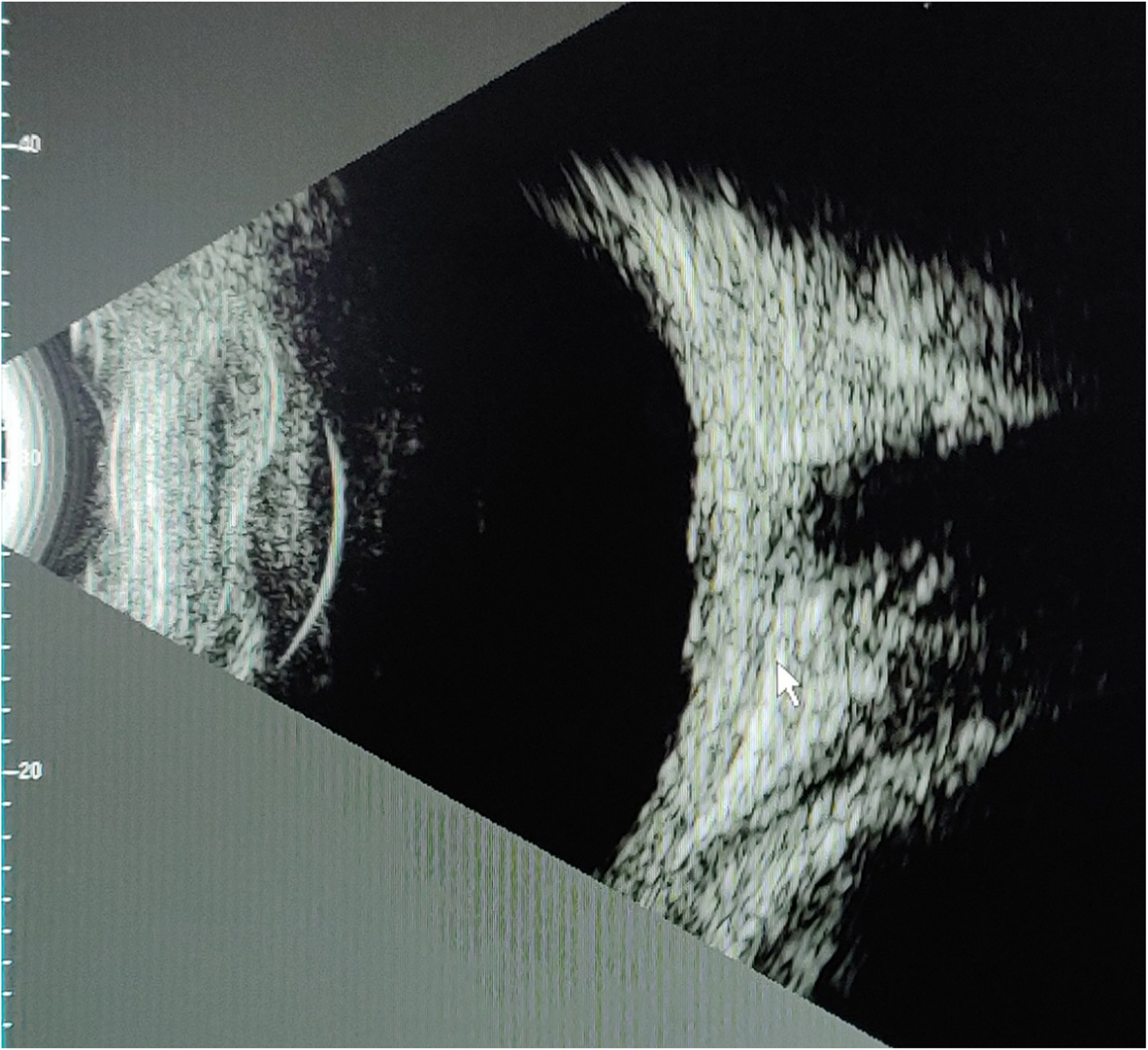
B scan picture showing “T sign” along with increased thickness of sclerochoroidal complex (2.62 mm)

A 9-year-old girl presented with complaints of redness in both the eyes and blurring of vision in the left eye for 1 month. There was no history of loss of appetite/weight, joint pains, cough, expectoration, or fever. There was no relevant contact history with TB case. She gave no history of hearing loss, headache or stiffness of neck, and premature greying of hair. Her immunization history was complete including BCG vaccine at birth. On general physical examination, her body weight was 28.8 Kg and height was 125 cm. Per abdomen palpation was soft with no signs of any organomegaly. On ocular examination, the best corrected visual acuity (BCVA) was 6/6, N6 in the right eye and 6/36, N36 in the left eye. She was orthophoric. Slit lamp examination showed nodular non-necrotizing anterior scleritis with localized deep conjunctival congestion at 9’o clock in the right eye and two localized nodular anterior scleritis lesions at 12’o clock and 8.30’ clock hour in the left eye. Anterior chamber was quiet in the right eye and a limbal phlycten was noted at 1’o clock with posterior synechiae and AC cells 2+ in the left eye (Fig. 1a, b). There were absence of mutton fat or fine keratic precipitates(KP’s) in AC. Non-contact tonometer recorded intraocular pressure of 12 mmHg in both the eyes. Fundus examination was normal in the right eye, and the left eye showed hyperemic disc edema with tortuous retinal vessels and pockets of subretinal fluid at the posterior pole. (Fig. 1c, d). Optical coherence tomography (OCT) revealed multiple pockets of neurosensory detachments with boggy swelling of retinal layers in the left eye (Fig. 1e, f). B-scan ultrasonography of the left eye showed thickening of choroid (2.62 mm) with presence of subtenon’s fluid (“classic T-sign”), hence confirming the diagnosis of posterior scleritis (Fig. 2). There was no evidence of posterior uveitis.

 Blood investigations showed normal hemoglobin, total leucocyte count, and differential leucocyte count. Erythrocyte sedimentation rate (ESR) was 22 mm in the first hour. Mantoux test was highly positive (22 mm × 20 mm) with a nodulo-ulcerative response. VDRL test was non-reactive and HIV1 and HIV2 were negative on ELISA test. CECT-Chest showed multiple discrete and conglomerating heterogeneously enhancing few necrotic superior mediastinal, pretracheal, paratracheal, carinal, prevascular, and bilateral hilar lymph nodes with few punctate calcifications suggestive of granulomatous etiology.

Based on clinical, hematological, and radiological evidences, the diagnosis of tuberculous non-necrotizing nodular anterior scleritis in both eyes with posterior scleritis in the left eye was made. Anti-tubercular treatment was initiated in consultation with a pediatrician. Intensive therapy was started with 4 drugs—isoniazid(H) @300 mg/day, rifampicin(R) @300 mg/day, pyrizinamide(Z) @750 mg/day, and ethambutol(E) @600 mg/day for 2 months followed by maintenance therapy with 2 drugs—isoniazid and rifampicin for 7 months. Oral prednisolone was administered @1 mg/kg body weight, starting from 30 mg and tapered slowly every 5 mg bi-weekly. The course of oral steroids was completed over 3 months. Liver function tests every three weekly was advised. Topical prednisolone acetate (1%) eight times a day with atropine (1%) three times a day was started in the left eye.

**Fig. 3:**
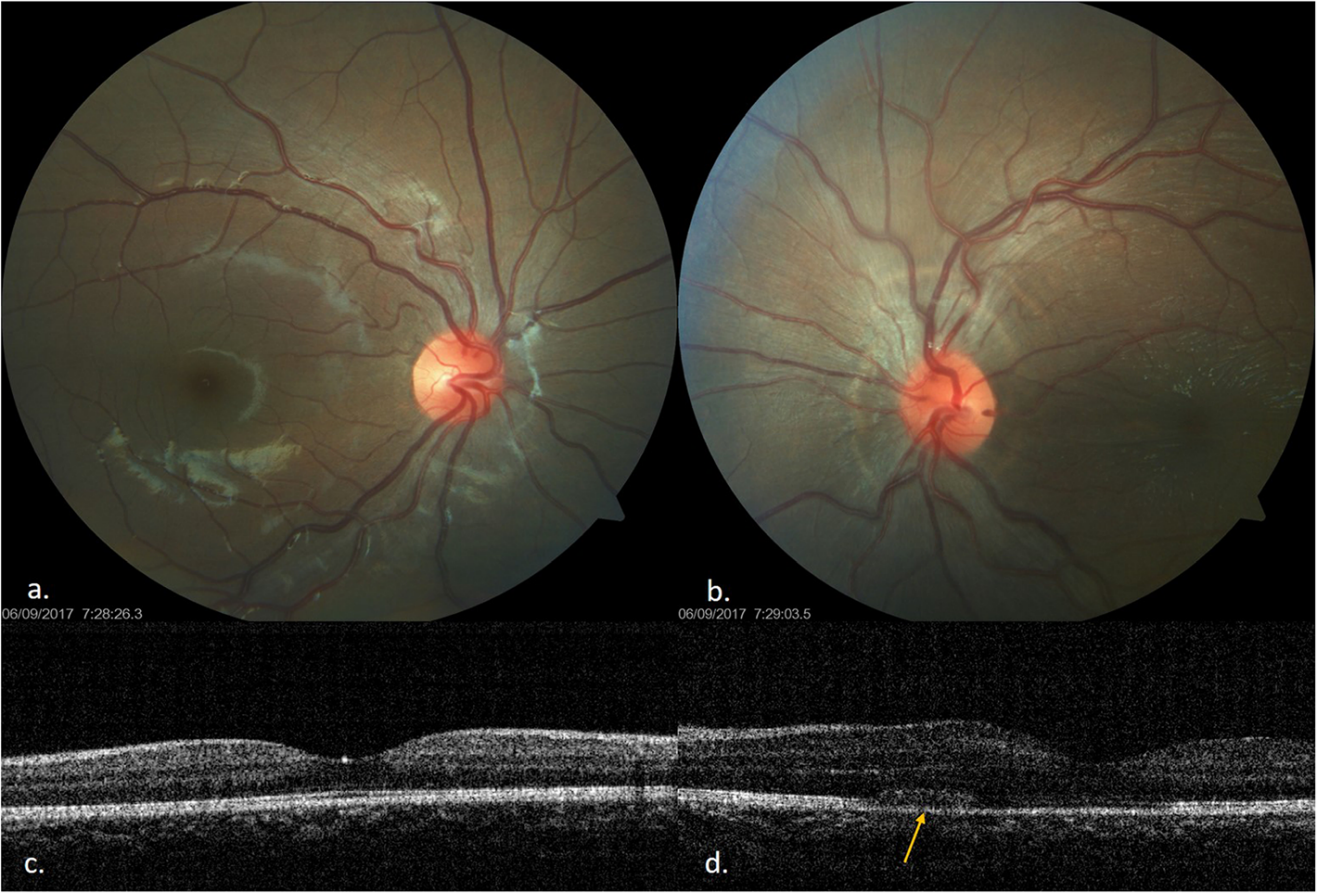
Follow-up 2 weeks. **a** Normal right eye fundus. **c** OCT of right eye. **b** Resolved disc edema and residual retinal striae. **d** OCT also shows decrease in retinal thickness, mild swelling of outer retinal layers with complete absorption of sub retinal fluid

**Fig. 4:**
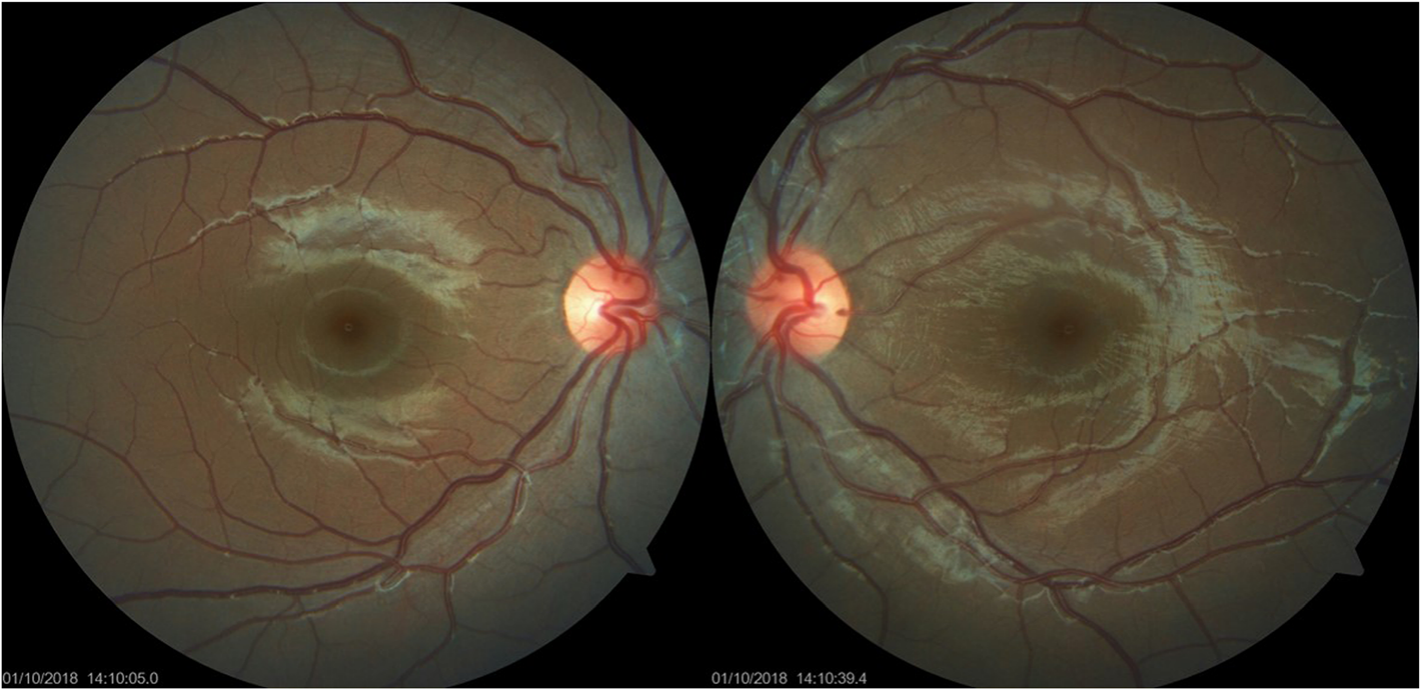
Final follow-up at 15 months: normal fundus photograph of both eyes

Follow-up at 1 week, the BCVA was 6/6, N6 in the right eye and 6/18, N24 in the left eye. There was decrease in the conjunctival congestion in the both eyes, and the disc hyperemia and retinal folds in the left eye also showed improvement. She was continued on the same treatment. At 2 months, the BCVA was 6/6, N6 in the both eyes with complete resolution of scleritis (Fig. 3a–d). She was continued with maintenance therapy of ATT for 7 months. Final follow up at 15 months showed complete resolution of disc edema with normal retinal architecture (Fig. 4)

## Discussion

Scleritis is the chronic, painful inflammation of the sclera, which can be anterior or posterior. Posterior scleritis is the inflammation of sclera posterior to insertion of the recti muscles. It can be idiopathic, infectious or autoimmune. Pediatric VKH is usually a diagnosis of exclusion after thorough clinical, systemic, and negative investigational workup. Incidence of scleritis due to infectious etiology has been estimated to be 7–8% of all scleritis cases in adults, and herpes zoster virus has been implicated as the most common cause of infectious scleritis [1]. Tuberculosis is a common infectious cause of scleritis especially in a TB endemic region. There are few case series and case reports about posterior scleritis in children in literature, but the exact incidence is still unknown. Hence, it can be stated that, scleral inflammation is rare in children. The incidence of pediatric scleritis in the recent study was 1.2% of all scleritis cases [2]. In case series by Majumdar et al., three patients had tuberculosis scleritis—only one had bilateral necrotizing scleritis with posterior scleritis and two had nodular anterior scleritis [2]. Majority of the reported cases of scleral involvement in tuberculosis is nodular anterior scleritis [3, 4]. Cheung and Chee observed concurrent anterior uveitis in 75% of the patients with posterior scleritis. In their article, they had only one case of tubercular posterior scleritis in a 19-year-old patient which required concurrent anti tubercular treatment [5].

The etiopathogenesis of tuberculous scleritis is believed to be due to direct invasion of M. tuberculosis bacteria or a result of antibody-mediated inflammatory reaction against M. tuberculosis bacilli proteins [6]. The most commonly reported scleritis type is nodular anterior scleritis as reported in literature. In a case series from Japan, Mantoux test positivity was highest in subgroup of patients with nodular scleritis (55.5%). Our case had a fulminant ulcerative response to 5 Tuberculin unit (TU) PPD in 48 h, which strongly supported our diagnosis along with enlarged mediastinal lymphadenopathy and necrosis within it on contrast enhanced CT scan (CECT-chest). Cornea or anterior chamber was not involved, unlike few cases which had concurrent PUK lesions in the affected eyes [7].

Posterior scleritis in children secondary to TB is very rare and has plethora of clinical manifestations. It is often misdiagnosed as some other clinical entity. It can present with severe inflammation, orbital involvement with lid signs, and restriction of extraocular muscle movement making the diagnosis more difficult especially in children [8]. Diagnosis of posterior scleritis was made according to the scheme proposed by Watson and Hayreh, using a combination of clinical findings and demonstration of scleral thickening and presence of “T-sign” on B-scan ultrasonography [9].

Clinical signs and symptoms usually resolve completely with intravenously or orally administered high-dose corticosteroid along with concurrent anti-tubercular medications if TB is the causative agent. Posterior scleritis in children, even in its severe form, has a good visual prognosis with the commencement of early appropriate treatment. Pain may or may not be associated with pediatric posterior scleritis. However, the absence of pain does not exclude the posterior scleritis [8].

Our case illustrates that posterior scleritis can present with acute redness, reduced vision with no pain as a predominant feature. As it is a rare but treatable condition which can have potentially vision threatening serious consequences, a high index of suspicion is necessary to make the diagnosis in pediatric population. She is the youngest girl diagnosed with TB as the cause of posterior scleritis in literature till date. The report also demonstrates the pathognomonic features of posterior scleritis on B-scan ultrasound.

## Data Availability

All data generated or analyzed during this study are included in this published article.

## Abbreviations

AC: Anterior chamber
ATT: Anti tubercular treatment
BCG: Bacillus Calmette-Guérin
BCVA: Best corrected visual acuity
CECT: Contrast enhanced computed tomography
ESR: Erythrocyte sedimentation rate
PUK: Peripheral ulcerative keratitis
TB: Tuberculosis
TU: Tuberculin unit

## References

[1] Jabs DA, Mudun A, Dunn JP (2000). Episcleritis and scleritis: clinical features and treatment results. Am J Ophthalmol.

[2] Majumder PD, Ali S, George A, Ganesh S (2018). Clinical profile of scleritis in children. Ocul Immunol Inflamm.

[3] Sharma R, Marasini S, Nepal BP (2010). Tubercular scleritis. Kathmandu Univ Med J.

[4] Yadav S, Rawal G (2015). Tubercular nodular episcleritis: a case report. J Clin Diagn Res.

[5] Cheung CM, Chee SP (2012). Posterior scleritis in children: clinical features and treatment. Ophthalmology.

[6] Bloomfield SE, Mondino B, Gray GF (1976). Scleral tuberculosis. Arch Ophthalmol.

[7] Arora T, Sharma N, Shashni A (2015). Titiyal JS Peripheral ulcerative keratitis associated with chronic malabsorption syndrome and miliary tuberculosis in a child. Oman J Ophthalmol.

[8] Woon WH, Stanford MR, Graham EM (1995). Severe idiopathic posterior scleritis in children. Eye.

[9] Watson PG, Hayreh SS (1976). Scleritis and episcleritis. Br J Ophthalmol.

